# Correlations between [^68^Ga]Ga-DOTA-TOC Uptake and Absorbed Dose from [^177^Lu]Lu-DOTA-TATE

**DOI:** 10.3390/cancers15041134

**Published:** 2023-02-10

**Authors:** Ragnar Bruvoll, Johan Blakkisrud, Lars Tore Mikalsen, James Connelly, Caroline Stokke

**Affiliations:** 1 Division of Radiology and Nuclear Medicine, Oslo University Hospital, 0424 Oslo, Norway; 2 Department of Physics, University of Oslo, 0315 Oslo, Norway; 3 Department of Life Sciences and Health, Oslo Metropolitan University, 0130 Oslo, Norway

**Keywords:** peptide receptor radionuclide therapy, [^68^Ga]Ga-DOTA-TOC, PET/CT, [^177^Lu]Lu-DOTA-TATE, SPECT/CT, dosimetry, prediction model

## Abstract

**Simple Summary:**

Peptide receptor radionuclide therapy (PRRT) with [^177^Lu]Lu-DOTA-TATE is used to treat gastroenteropancreatic neuroendocrine tumours (GEP-NET) by targeting somatostatin receptors (SSTRs) found on the cell surface. High SSTR expression on [^68^Ga]Ga-DOTA-TOC PET/CT decides patient eligibility. This study aimed to investigate potential correlations between therapeutic absorbed dose to tumours and pre-therapeutic [^68^Ga]Ga-DOTA-TOC uptake, and found that the [^68^Ga]Ga-DOTA-TOC PET imaging may predict the [^177^Lu]Lu-DOTA-TATE uptake to a certain extent. However, there could be a high variance between predicted and actual absorbed doses for individual patients.

**Abstract:**

Purpose: The aim of this paper was to investigate correlations between pre- therapeutic [^68^Ga]Ga-DOTA-TOC uptake and absorbed dose to tumours from therapy with [^177^Lu]Lu-DOTA-TATE. Methods: This retrospective study included 301 tumours from 54 GEP-NET patients. The tumours were segmented on pre-therapeutic [^68^Ga]Ga-DOTA-TOC PET/CT, and post-therapy [^177^Lu]Lu-DOTA-TATE SPECT/CT images, using a fixed 40% threshold. The SPECT/CT images were used for absorbed dose calculations by assuming a linear build-up from time zero to day one, and mono-exponential wash-out after that. Both SUV_mean_ and SUV_max_ were measured from the PET images. A linear absorbed-dose prediction model was formed with SUV_mean_ as the independent variable, and the accuracy was tested with a split 70–30 training-test set. Results: Mean SUV_mean_ and SUV_max_ from [^68^Ga]Ga-DOTA-TOC PET was 24.0 (3.6–84.4) and 41.0 (6.7–146.5), and the mean absorbed dose from [^177^Lu]Lu-DOTA-TATE was 26.9 Gy (2.4–101.9). A linear relationship between SUV_mean_ and [^177^Lu]Lu-DOTA-TATE activity concentration at 24 h post injection was found (R^2^ = 0.44, *p* < 0.05). In the prediction model, a root mean squared error and a mean absolute error of 1.77 and 1.33 Gy/GBq, respectively, were found for the test set. Conclusions: There was a high inter- and intra-patient variability in tumour measurements, both for [^68^Ga]Ga-DOTA-TOC SUVs and absorbed doses from [^177^Lu]Lu-DOTA-TATE. Depending on the required accuracy, [^68^Ga]Ga-DOTA-TOC PET imaging may estimate the [^177^Lu]Lu-DOTA-TATE uptake. However, there could be a high variance between predicted and actual absorbed doses.

## 1. Introduction

Gastroenteropancreatic neuroendocrine tumours (GEP-NET) are heterogeneous malignancies, characterised by a variable expression of somatostatin receptors (SSTR) on the cell surface of the dispersed neuroendocrine system in the pancreas and other parts of the gastrointestinal tract [1,2,3]. Peptide receptor radionuclide therapy (PRRT) is a molecular cancer treatment using SSTR to target GEP-NETs. A high uptake of a diagnostic somatostatin analogue (SSTA) in tumour lesions is a common measure of PRRT eligibility [4,5]. ^68^Ga-DOTA-labelled SSTAs are currently considered the preferred tool for patient evaluation of SSTR expression [6,7,8,9], and, for example, the PET tracer [^68^Ga]Ga-DOTA-TOC has shown favourable performance and is commonly used [10].

Several radiolabelled SSTA alternatives, such as [^111^In]In-DOTA-TOC, [^111^In]In-octreotide, [^90^Y]Y-DOTA-TOC, [^177^Lu]Lu-DOTA-NOC, [^177^Lu]Lu-DOTA-TATE, and [^177^Lu]Lu-DOTA-TOC, have been applied for therapeutic use [8,11,12,13,14,15]. PRRT with [^177^Lu]Lu-DOTA-TATE delivers targeted radiation and has shown promising response and survival statistics [15,16], while additionally permitting post-treatment imaging and personalised dosimetry [17]. [^177^Lu]Lu-DOTA-TATE is usually prescribed by multiple cycles of 7.4 GBq with intervals of eight weeks between [15]. Based on post-therapy SPECT/CT measurements of activity concentration at various time points, absorbed dose calculations can be performed. Dosimetry can be used for treatment optimisation, by limiting absorbed dose to organs at risk, and should ideally be performed for tumours as well as to monitor the absorbed dose to the target tissue. A high inter- and intra-patient variability in tumour-absorbed doses from PRRT is commonly observed [18,19,20], emphasising the need for personalised treatment. Given a sufficiently correlated uptake, the absorbed dose from PRRT with [^177^Lu]Lu-DOTA-TATE could potentially be predicted from measurements of [^68^Ga]Ga-DOTA-TOC. While the two radiopharmaceuticals are based on similar peptides, differences in SSTR affinity between TOC and TATE have been observed in vitro, although the clinical consequences of these differences remain uncertain [21]. In comparisons of tumour uptake of [^68^Ga]Ga-DOTA-TOC and [^68^Ga]Ga-DOTA-TATE as a tool for staging and PRRT patient selection, only minor clinical implications were shown [10,22]. [^177^Lu]Lu-DOTA-TATE has been found to have a 2.1 longer residence time in tumours than [^177^Lu]Lu-DOTA-TOC [23], indicating that, opposed to the uptake level, the pharmacokinetics vary. Still, these comparisons are not directly translatable to predict correlations between [^68^Ga]Ga-DOTA-TOC and [^177^Lu]Lu-DOTA-TATE, as the radionuclide can possibly also change the molecular behaviour. A few earlier studies have investigated the correlation between PET-derived [^68^Ga]Ga-DOTA-TOC uptake and SPECT-derived [^177^Lu]Lu-DOTA-TATE uptake [24], and one study also investigated and found potential for absorbed dose prediction [25].

The aim of this work was to explore the correlation between [^68^Ga]Ga-DOTA-TOC SUV and [^177^Lu]Lu-DOTA-TATE uptake parameters in the first of the standard 7.4 GBq treatment fractions for tumours, and to investigate an absorbed dose prediction model for the therapy based on pre-therapeutic [^68^Ga]Ga-DOTA-TOC PET/CT scans. 

## 2. Materials and Methods

### 2.1. Patient Population

This study included 54 consecutive patients diagnosed with metastatic and progressive or recurrent GEP-NET, treated with at least one fraction of [^177^Lu]Lu-DOTA-TATE PRRT. Eligibility criteria for the treatment were radiologic, symptomatic or biochemical progression despite optimal conventional treatment, and a high expression of SSTR as assessed on [^68^Ga]Ga-DOTA-TOC PET/CT scans. Histological sampling was performed prior to the treatment to establish tumour grade and Ki-67 index. Adequate hepatic, renal and hematologic function are also critical prerequisites for treatment.

### 2.2. Image Acquisition

**Figure 1:**
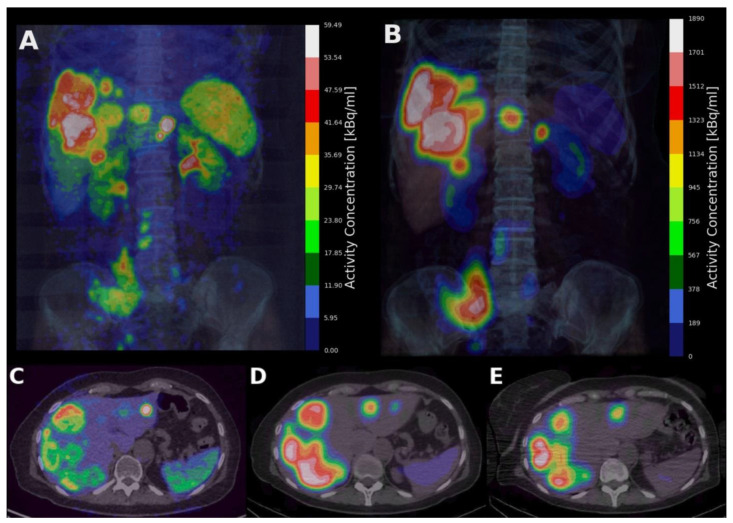
Maximum intensity projection (MIP) of a [^68^Ga]Ga-DOTA-TOC PET/CT image (**A**); and corresponding [^177^Lu]Lu-DOTA-TATE SPECT/CT image at the 24 h p.i. timepoint (**B**); axial slices of [^68^Ga]Ga-DOTA-TOC (**C**); and [^177^Lu]Lu-DOTA-TATE at t_24_ (**D**); and t_168_ (**E**), showing uptake in SSTR-expressing tumours. Physiologic uptake in kidneys, spleen and liver is also visible.

Pre-therapeutic PET/CT imaging (example in Figure 1A,C) was performed on a GE Discovery MI, 78 min (range: 54–96 min) post administration a nominal, fixed, activity dosage of 150 MBq [^68^Ga]Ga-DOTA-TOC for patients above 35 years old. The acquisition time was 100 s per bed with 50% overlap. The images were gated using the Varian trigger system and reconstructed in the expiratory phase (Q.Static). The images were reconstructed with a Bayesian penalised likelihood reconstruction (Q.Clear) with a beta factor of 500, on a 384 × 384 matrix of 1.82 mm pixels with a slice thickness of 2.79 mm. The CT scans were acquired at 120 kV and a dose modulation noise index of 34.5 and reconstructed with an iterative reconstruction (ASIR-V) with 40% filtering on 512 × 512 matrices. The pixel size was 0.84 mm with slice thickness 0.625 mm.

[^177^Lu]Lu-DOTA-TATE SPECT/CT imaging (example in Figure 1B,D,E) was on average performed 23.1 h (range 21.0–26.8) and 167.0 h (range 119.1–218.8 h) post administration (t_24_ and t_168_, respectively) of the first treatment cycle for all patients. An additional image (t_4_) on average 4.7 h (range 3.4–6.5) was acquired for nine of the patients. The average administered activity was 7574 MBq (range 7247–7907 excl. one patient given 4195 MBq). The imaging was performed on a GE Discovery 670 NM/CT scanner with a medium energy, general purpose collimator. The images were acquired with an energy window set at 208 keV +/− 10% with 120 projections of 30 s frame duration, and two adjacent scatter windows of +/−5%. A scatter- and attenuation-corrected reconstruction with four iterations and eight subsets without resolution recovery was performed with the vendor software (Xeleris, GE Healthcare). Matrix size was 128 × 128 and isotropic voxels of 4.42 mm were reconstructed. The CT scans were acquired at 120 kV and a dose modulation noise index of 26 and reconstructed with an iterative reconstruction (ASIR) with 40% filtering on 512 × 512 matrices. The pixel size was 0.98 mm with slice thickness 2.5 mm.

### 2.3. [68Ga]Ga-DOTA-TOC PET Measurements

Tumours which could be visually matched between all images (PET and SPECT at t_24_ and t_168_), with negligible overlap with adjacent activity sources, were included for analysis. A manual identification of tumours confirmed by a nuclear medicine specialist was followed by a fixed threshold method for measurements, using a threshold of 40% [24] of the SUV_max_. Both SUV_max,_ SUV_mean,_ and the volume defined by the threshold, referred to as the somatostatin receptor expressing tumour volume (SRETV) from here on, were recorded.

### 2.4. [177Lu]Lu-DOTA-TATE SPECT Measurements and Tumour Dosimetry

The tumours were delineated using 40% threshold determined by volume matching in a phantom study, from which recovery coefficients were also found (Supplementary Figure S1). The VOI-derived counts were converted to activity by a system specific calibration coefficient of 8.4 cps/MBq. Time-activity curves (TACs) were calculated by assuming a linear activity build-up from time of administration to the first measuring point (t_24_), followed by a mono-exponential curve fit based on t_24_ and t_168_. The TAC was integrated to yield the time-integrated activity (*Ã*). The tumour mass (*m*) was estimated by measuring the volume at t_24_ and assuming a tissue mass density of 1 g/mL. The absorbed dose was calculated by assuming local deposition and a dose factor *DF* = 0.0853 Gy∙g/MBq∙h, including all non-penetrative radiation from ^177^Lu reported in the ICRP 2008 publication [26]. The activity concentrations at each time point, $A_{conc,4}$, $A_{conc,24}$, and $A_{conc,168}$ were also calculated and normalised for injected amount of activity to the individual patient for some of the analyses.

(1) $$D=\frac{\tilde{A}}{m}DF$$

### 2.5. Prediction Analyses

A linear estimator between [^68^Ga]Ga-DOTA-TOC *SUV_mean_* and [^177^Lu]Lu-DOTA-TATE absorbed dose per administered activity (*D/A_adm_*) was used.

(2) $$D/A_{adm}=SUV_{mean}\cdot \alpha +\beta$$

Here *α* and *β* are fitting parameters.

To test the performance of the predictive model, the patients were split into a 70–30 training (37 patients, 210 tumours) test (17 patients, 91 tumours) set. The root mean squared error the mean absolute error and the coefficient of determination (R^2^) were calculated for the test set based on fitting parameters found from the training set.

The prediction model was further explored by testing the ability to classify the tumour absorbed dose as higher or lower than the population median using logistic regression and receiver operating characteristics curves.

### 2.6. Group Analysis

Differences in absorbed dose and differences between predicted and actual absorbed dose were investigated for multiple tumour groups. Tumours were grouped by anatomical site (hepatic, lymphatic and skeletal lesions were compared), SUV-parameters and tumour mass. Two approaches were explored; one where tumours were divided into groups of equal size, and one where equal range of the parameter were grouped. The group differences of relative error between predicted and measured absorbed dose were tested with a one-way analysis of variance (ANOVA)-test and significance of individual group differences were conducted using the Tukey-test. The difference of absorbed dose between groups was also tested.

### 2.7. Statistics

A Shapiro–Wilk normality test and visual inspection of the probability plots of the residues were performed to test for normality. Pearson correlation was performed to test linear correlation between parameters. The linear correlation was evaluated in terms of the coefficient of determination, R^2^. Mean absolute error and root mean squared error were also used to score model performance. A significance level of 0.05 was used. All statistical analyses were conducted with the SciPy statistical functions module and the Sklearn library, both in Python 3.8 (Python Software Foundation, Wilmington, DE, USA). The probability plot of the residuals was created with the SciPy stats probplot function against the normal distribution.

## 3. Results

### 3.1. Patient Characteristics and Tumour Parameters

**Figure 2:**
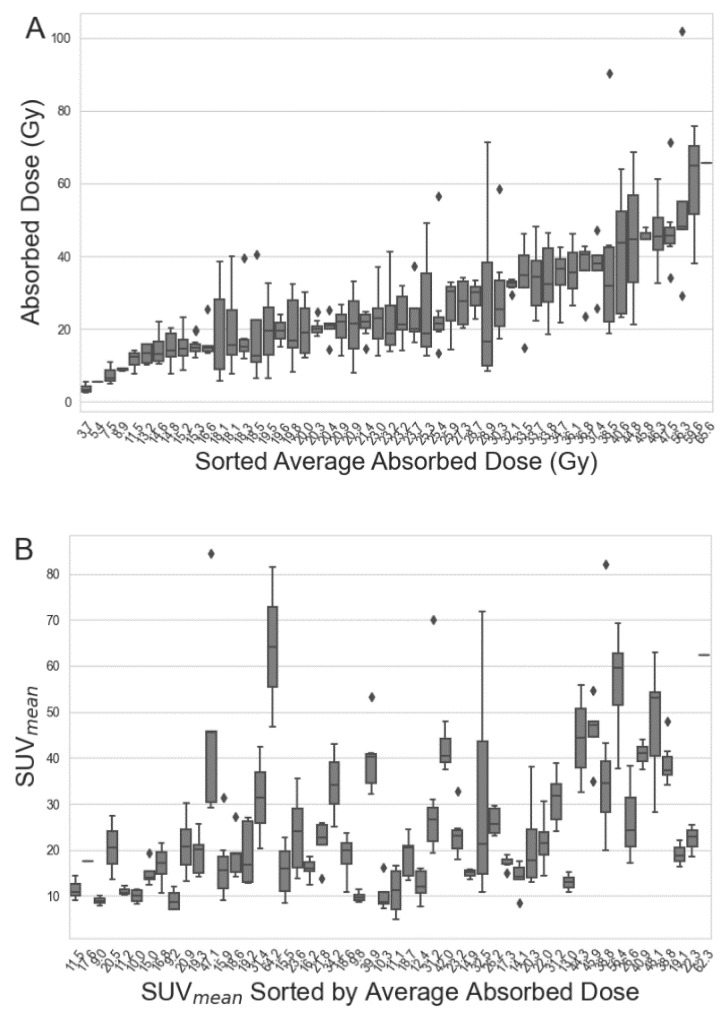
Box-and-whisker plots of [^177^Lu]Lu-DOTA-TATE absorbed dose (**A**); and [^68^Ga]Ga-DOTA-TOC SUV_mean_ (**B**), representing all the analysed tumours in each patient. The patients are sorted by average tumour-absorbed dose in both figures.

This study included 54 patients (15 female, 39 male) with an average age of 65 years (range 38–82 years). A total of 301 tumours (1–15 per patient, 3 being most frequent) were analysed, the majority being hepatic (230/301) or lymphatic (35/301). The [^68^Ga]Ga-DOTA-TOC and [^177^Lu]Lu-DOTA-TATE parameters are included in Table 1 and Figure 2.

### 3.2. Correlation between [^68^Ga]Ga-DOTA-TOC and [^177^Lu]Lu-DOTA-TATE Parameters for the Full Data Set

**Figure 3:**
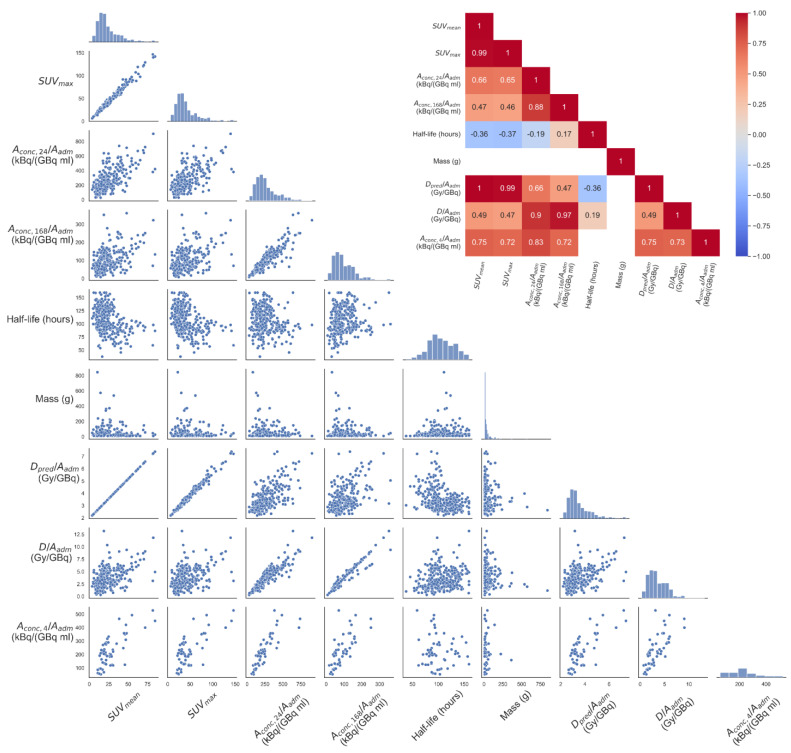
All parameters shown as scatter plots and their distribution on the diagonal. The inset heatmap shows the Pearson correlation coefficient where a significant linear correlation between parameters was found. The full data set is shown, and the predicted absorbed doses are based on the full data set.

The absorbed dose had a strong linear correlation to $A_{conc,24}$ (R^2^ = 0.82, *p* < 0.05) but not to the effective half-life after 24 h (R^2^ = 0.04, *p* < 0.05) (Figure 3). SUV_mean_ and SUV_max_ were strongly correlated (R^2^ = 0.98, *p* < 0.05). A linear correlation was found between SUV and $A_{conc,4}/A_{adm}$ (SUV_mean_: R^2^ = 0.57, *p* < 0.05, SUV_max_: R^2^ = 0.53, *p* < 0.05). A somewhat weaker correlation was observed between SUV and $A_{conc,24}/A_{adm}$ (SUV_mean_: R^2^ = 0.44, *p* < 0.05, SUV_max_: R^2^ = 0.42, *p* < 0.05). The linear correlation between SUV-parameters and absorbed dose was weak (SUV_mean_: R^2^ = 0.25, *p* < 0.05, SUV_max_: R^2^ = 0.23, *p* < 0.05). The distribution of residuals of the data was deemed normal by visual inspection of the probability plots (Supplementary Figure S2).

### 3.3. Absorbed Dose Prediction

**Figure 4:**
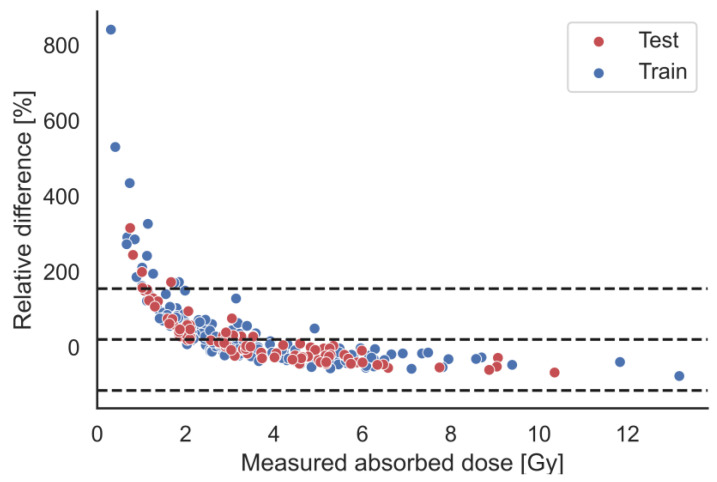
Relative error between predicted and calculated absorbed dose. The central dashed line is the average bias and the lower and upper 95% confidence intervals for the test are marked. Colours indicate whether the data point is from the training- or test-set. The predicted absorbed doses are calculated from parameters found in the training data set.

A prediction model based on the linear correlation between SUV_mean_ and $D/A_{adm}$ was explored. The mean absolute error and root mean squared error in the test-set was 1.33 and 1.77 Gy/GBq respectively. The R^2^-value of the test set was 0.24. Model parameters were α=0.064 and β=1.96. The relative error ($\Delta_{AD}$) between absorbed dose estimated by the prediction model (D_pred_) and the actual absorbed dose is shown in Figure 4.

**Figure 5:**
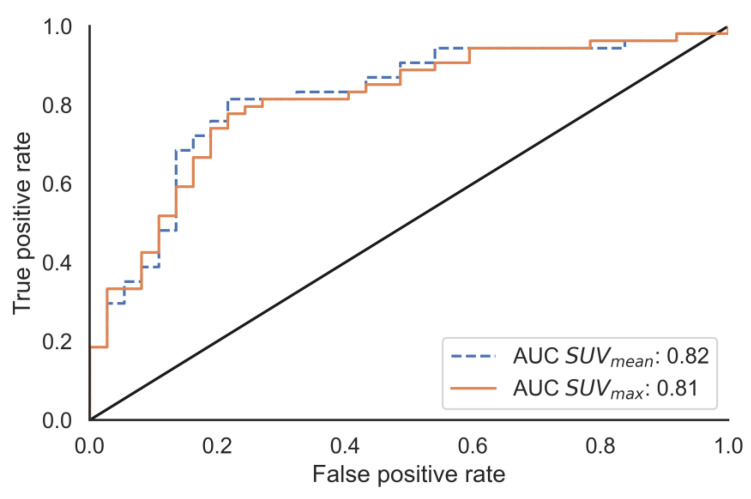
Receiver operating characteristic analysis. The ability to classify the absorbed dose as either above or below the test population median value of 3.0 Gy/GBq was tested.

Using the population median absorbed dose, 23.3 Gy, as the cut-off point of a binary classifier, the predictive ability of the model was further tested. The performances of SUV_mean_ and SUV_max_ were comparable according to the ROC analysis (Figure 5).

### 3.4. Tumour Sub-Groups

**Figure 6:**
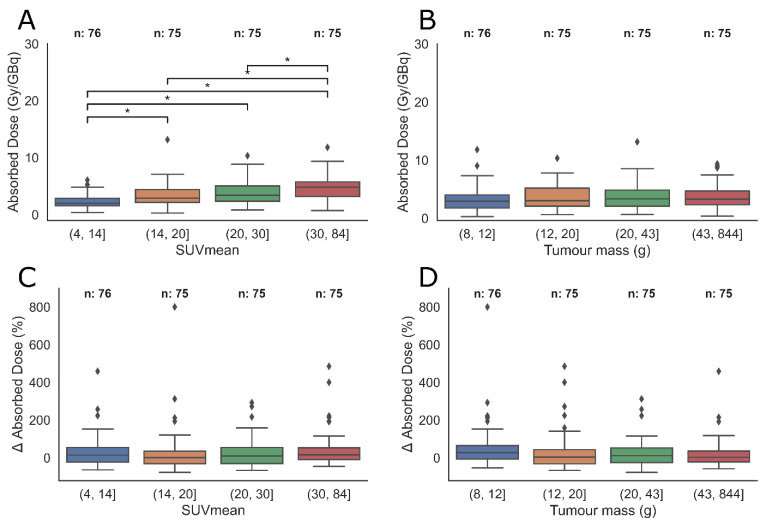
Differences of absorbed dose (**A**,**B**); and relative difference between predicted and calculated absorbed dose (**C**,**D**) between tumours grouped by SUV_mean_ and tumour mass. Grouping was done by keeping group sizes equal. Stars indicate significant differences of the group means (Tukey test). Only the absorbed dose of tumours grouped by SUV_mean_ was different between groups. A grouping based on equal ranges of mass and SUV_mean_-values is found in the Supplementary Materials (Supplementary Figure S3).

None of the anatomical site groups showed significant inter-group differences. Grouping the tumours by SUV-values did reveal that a higher absorbed dose was observed in patients with higher SUV-values (Figure 6A). There were no statistical differences between tumours grouped by tumour mass. The results of the group analysis where an equal range of the parameters are used are found in Supplementary Figure S3.

## 4. Discussion

This retrospective study of 54 patients with GEP-NET found a weak albeit significant correlation between tumour specific uptake of [^68^Ga]Ga-DOTA-TOC and [^177^Lu]Lu-DOTA-TATE. There was a high inter- and intrapatient variability in tumour measurements both for [^68^Ga]Ga-DOTA-TOC and [^177^Lu]Lu-DOTA-TATE. An absorbed dose prediction model based on SUV_mean_ was explored and assessed, showing that there could be a high deviation between predicted and actual absorbed dose, especially for tumours with lower absorbed doses.

Previous publications have reported median [^68^Ga]Ga-DOTA-TOC SUV_mean_ for tumours ranging from approx. 10 to 25 and SUV_max_ ranging from approx. 20 to 40 [24,25,27,28], which is partly lower than our measurements. Slight variations may be due to differences in scanner performance, imaging acquisition (e.g., use of respiratory gating) and reconstruction settings. While delineation techniques will also be important for SUV_mean_, this should not affect the SUV_max_ measurements. The absorbed dose previously found for [^177^Lu]Lu-DOTA-TATE ranges from a few to 170 Gy [18,19,20,29,30,31] which is comparable to our measurements. It should be noted that some of the previous publications report on more or less heterogeneous groups of patients with regard to types of NET than those reported in the current work. Still, the overall result of our absorbed dose calculations is in agreement with the ones previously found in somatostatin-receptor expressing NETs.

As described in the introduction, a few studies have reported on attempts to correlate diagnostic and post therapeutic imaging of GEP-NETs. One group did a feasibility study on a small, mixed population and investigated SUV-parameters defined with a 40% threshold on both [^68^Ga]Ga-DOTA-TOC images and 24 h p.i. images of [^177^Lu]Lu-DOTA-TATE [24]. They still found a significant but, commented by the authors, imperfect correlation. Another group looked at the correlation between PET-images of [^68^Ga]Ga-DOTA-TOC and [^177^Lu]Lu-Octreotate absorbed doses calculated from planar scans [25] of 61 tumours in 21 patients. They similarly found a significant but somewhat variable relationship between SUV_mean_ and absorbed dose. They argue that a “high SUV” (meaning SUV_mean_ > 15 and SUV_max_ > 25) could indicate a resulting “high dose range” of >10 Gy/GBq. The authors argued that caution had to be used as a considerable fraction of the “high” SUV tumours gave low absorbed doses. The group analysis in the current work shows the same tendencies, as a higher absorbed dose is associated with a higher SUV-value (Figure 6), but the large overlap of absorbed dose between SUV-groups still encourages caution. A limitation of the current study is the potential selection bias of only including tumours that could be clearly separated from adjacent tissues. While this probably results in more reliable measurements, and a broader range of tumours than selection of a predetermined number, especially tumours of low uptake may still be subjected to exclusion.

Our analysis show that absorbed dose from administration of [^177^Lu]Lu-DOTA-TATE cannot be accurately predicted based on a single diagnostic scan of [^68^Ga]Ga-DOTA-TOC performed in current clinical routine at our institution. A large intra- and interpatient variation was demonstrated by the high relative error variance, most notably for tumours of low absorbed dose where the predicted absorbed dose is highly overestimated (Figure 4). The cut-off at the population median absorbed dose was used as a starting point to explore a minimum ability for prediction. While the optimal cut-off value would be the absorbed dose that would classify the treatment as successful, this value is not firmly established. The median was therefore selected as a robust parameter, so the results could be compared with other tumour populations, as variations in methodology between centres will probably not affect the median to a large degree. Although SUV_mean_ was used in the current analyses, an argument can be made for the use of SUV_max_ as, although being sensitive to image noise, this parameter is virtually independent of the segmentation technique and is simple to define. In the current work, it was also shown to be highly correlated with SUV_mean_ (Figure 3), and while SUV_mean_’s similar interpretation to $A_{conc,24}/A_{adm}$ resulted in its use, the results would be highly similar for SUV_max_.

The absorbed-dose calculation that represents the ground truth in this work is primarily based on two parameters; the effective half-life for the mono-exponential wash-out phase, and the [^177^Lu]Lu-DOTA-TATE activity concentrations. The PRRT absorbed dose was highly correlated to the [^177^Lu]Lu-DOTA-TATE activity concentration at 24 and 168 h p.i., but considerable less correlated with the effective half-life after 24 h (Figure 3). Interestingly, the SUV_mean_ was negatively associated with the effective half-life of [^177^Lu]Lu-DOTA-TATE, and this relationship was actually also stronger than the one between effective half-life and absorbed dose. This leads to the systematic trend in Figure 4, which may make it appear as though a non-linear model would have been better suited for the prediction model. The negative association between SUV_mean_ and effective half-life of [^177^Lu]Lu-DOTA-TATE could be due to biological, or even radiobiogical, factors and would be interesting to pursue in further studies. While the SUV_mean_ was positively correlated with [^177^Lu]Lu-DOTA-TATE activity concentration at 24 and 168 h p.i. (Figure 3), for a subpopulation of the patients a SPECT/CT at 4 h was also obtained, and the $A_{conc,4}/A_{adm}$ had an even better correlation with SUV_mean_. This indicates that the delay between administration and image acquisition for [^68^Ga]Ga-DOTA-TOC and [^177^Lu]Lu-DOTA-TATE can possibly explain the poor linear correlation between SUV_mean_ of [^68^Ga]Ga-DOTA-TOC and $A_{conc,24}/A_{adm}$ of [^177^Lu]Lu-DOTA-TATE, and hence partly also the absorbed dose deviation. Furthermore, it can be considered whether a dose prediction model should include a separate term describing an association between SUV_mean_ and effective half-life of [^177^Lu]Lu-DOTA-TATE. It should also be pointed out that there may be various patient- or tumour-specific factors, not investigated in this work, that could also be incorporated in order to form an improved prediction model. 

The SPECTs obtained indicate that 168 h p.i. is a better time point for single time point tumour dosimetry than 24 h p.i. (R^2^-value of 0.82 and 0.94 for $A_{conc,24}/A_{adm}$ and $A_{conc,168}/A_{adm}$ respectively), in agreement with previous investigations [32,33]. Still, the possibility for delayed 24 h imaging of a PET tracer (possibly using another radionuclide) can prove fruitful for continued investigations.

## 5. Conclusions

There was a high inter- and intra-patient variability in tumour measurements both for [^68^Ga]Ga-DOTA-TOC and [^177^Lu]Lu-DOTA-TATE. While a significant correlation between tumour-specific uptake of [^68^Ga]Ga-DOTA-TOC and [^177^Lu]Lu-DOTA-TATE absorbed dose was found for individual tumours, the correlation was noisy, and the absorbed-dose prediction model based on SUV_mean_ should be used with care depending on the purpose and required accuracy.

## Figures and Tables

**Table 1:** Mean and standard deviation of parameters measured in all 301 tumours from [^68^Ga]Ga-DOTA-TOC PET/CT and [^177^Lu]Lu-DOTA-TATE SPECT/CT images.

	Total	Training Dataset	Test Dataset
Characteristic			
Number of patients	54	37	17
Number of tumours	301	210	91
PET parameters			
Mean ± Standard deviation			
SUV_mean_	24.0 ± 14.5	24.1 ± 15.1	23.7 ± 12.9
SUV_max_	41.0 ± 24.9	41.2 ± 26.0	40.7 ± 22.2
SRETV [mL]	29.4 ± 45.9	31.1 ± 49.8	25.5 ± 35.5
SPECT parameters			
Mean ± Standard deviation			
Absorbed dose [Gy]	26.9 ± 15.1	26.6 ± 15.1	27.6 ± 15.2
Absorbed dose/A_adm_ [Gy/GBq]	3.6 ± 2.0	3.5 ± 2.0	3.8 ± 2.0
$A_{conc,4}/A_{adm}$ [(kBq/mL)/GBq] *	224.8 ± 113.1	220.0 ± 92.3	235.1 ± 151.3
$A_{conc,24}/A_{adm}$ [(kBq/mL)/GBq]	262.3 ± 148.4	256.2 ± 148.2	276.3 ± 148.7
$A_{conc,\;168/A_{adm}}$ [(kBq/mL)/GBq]	99.0 ± 58.7	96.7 ± 60.4	104.2 ± 54.4
Effective half-life after t_24_ [hours]	105.7 ± 24.5	106.3 ± 23.7	104.3 ± 26.3
Tumour mass [g]	45.8 ± 81.8	45.3 ± 73.6	47.1 ± 98.5

* Not all patients underwent imaging at this time point, and only 57 tumours are represented by this number.

## Data Availability

The datasets generated during and/or analysed during the current study are available from the corresponding author on request.

## Supplementary Materials

The following supporting information can be downloaded at: https://www.mdpi.com/article/10.3390/cancers15041134/s1, Figure S1: An Esser phantom with spherical inserts of 2, 4, 8, 16 and 113 mL was used to determine threshold and recovery coefficients; Figure S2: Probability plots of the residuals of the predicted and calculated absorbed dose from SUV_mean_ (A) and SUV_max_ (B); Figure S3: The group analysis with equal group size instead of equal range in grouping parameters. Stars indicate statistically significant differences of the group means according to the Tukey-test.
