# Supplementary material for: Correlations between [68Ga]Ga-DOTA-TOC Uptake and Absorbed Dose from [177Lu]Lu-DOTA-TATE
Source: Cancers (Basel). 2023 Feb 10;15(4):1134. doi: 10.3390/cancers15041134 (PMC9954147; doi:10.3390/cancers15041134)
Supplement: Supplementary file 1 [file cancers-15-01134-s001.zip › cancers-2160924-supplementary.pdf]

Supplementary Material

This file is supplementary to "Correlations between [^{68}Ga]Ga-DOTA-TOC uptake and absorbed dose from [^{177}Lu]Lu-DOTA-TATE".

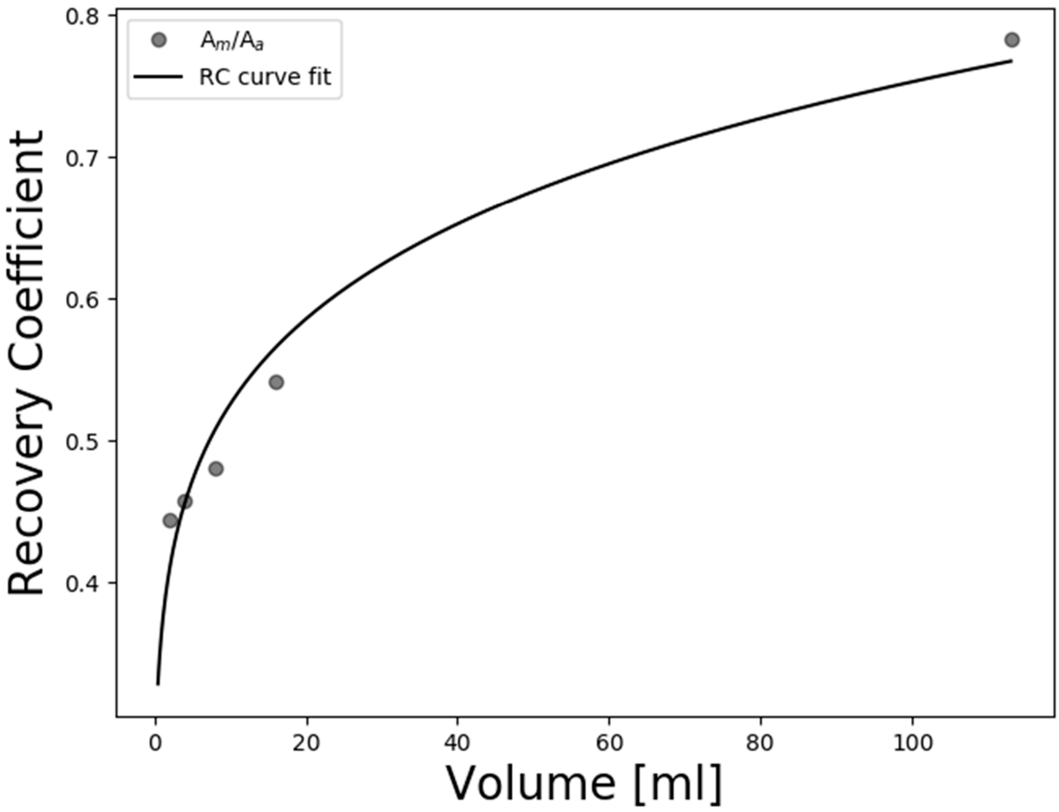

Supplementary figure S1: An Esser phantom with spherical inserts of 2, 4, 8, 16 and 113 ml was used to determine threshold and recovery coefficients. A threshold of 40 % was found to reproduce the volume of the inserts above 8 ml. Recovery coefficients (RCs) were derived from the same SPECT/CT phantom image by defining the ratio between measured (A_m) and actual (A_a) activity in the five spheres, using this 40% threshold. The RC curve was fitted to a volume (v) dependent sigmoid function $RC = 1/\left(1 + (\alpha/v)^{\beta}\right)$ with fit parameters α and β.

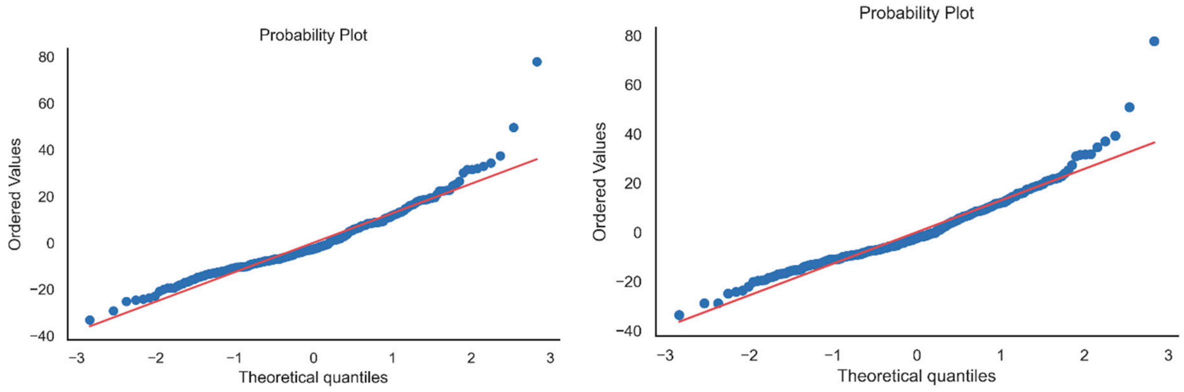

Supplementary figure S2: Probability plots of the residuals of the predicted and calculated absorbed dose from SUV_{mean} (A) and SUV_{max} (B).

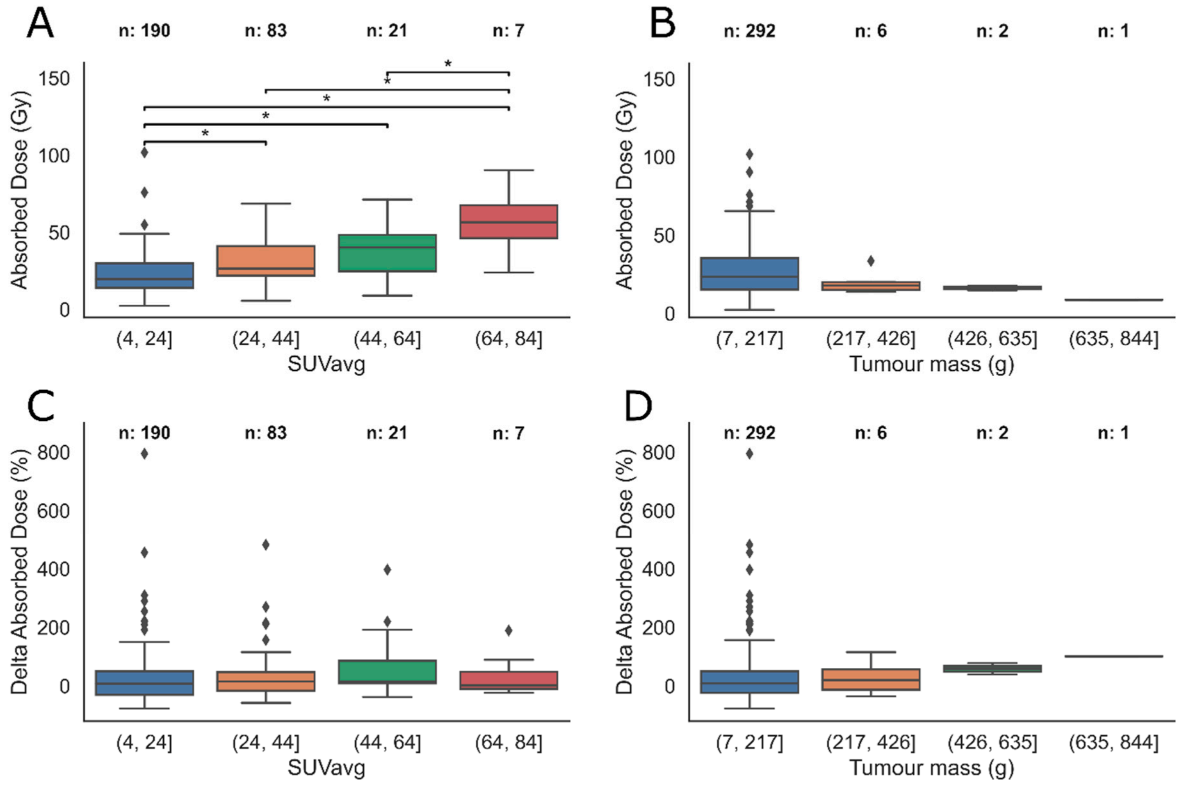

Supplementary figure S3: The group analysis with equal group size instead of equal range in grouping parameters. Stars indicate statistically significant differences of the group means according to the Tukey-test.
